# The Spatial Relationship between Apparent Diffusion Coefficient and Standardized Uptake Value of ^18^F-Fluorodeoxyglucose Has a Crucial Influence on the Numeric Correlation of Both Parameters in PET/MRI of Lung Tumors

**DOI:** 10.1155/2017/8650853

**Published:** 2017-12-17

**Authors:** Alexander W. Sauter, Bram Stieltjes, Thomas Weikert, Sergios Gatidis, Mark Wiese, Markus Klarhöfer, Damian Wild, Didier Lardinois, Jens Bremerich, Gregor Sommer

**Affiliations:** ^1^University Hospital Basel, University of Basel, Clinic of Radiology & Nuclear Medicine, Petersgraben 4, 4031 Basel, Switzerland; ^2^Diagnostic and Interventional Radiology, University Hospital Tübingen, Eberhard Karls University, Hoppe-Seyler-Straße 3, 72076 Tübingen, Germany; ^3^University Hospital Basel, University of Basel, Clinic of Thoracic Surgery, Spitalstrasse 21, 4031 Basel, Switzerland; ^4^Siemens Healthineers, Freilagerstrasse 40, 8047 Zürich, Switzerland

## Abstract

The minimum apparent diffusion coefficient (ADC_min_) derived from diffusion-weighted MRI (DW-MRI) and the maximum standardized uptake value (SUV_max_) of FDG-PET are markers of aggressiveness in lung cancer. The numeric correlation of the two parameters has been extensively studied, but their spatial interplay is not well understood. After FDG-PET and DW-MRI coregistration, values and location of ADC_min_- and SUV_max_-voxels were analyzed. The upper limit of the 95% confidence interval for registration accuracy of sequential PET/MRI was 12 mm, and the mean distance (*D*) between ADC_min_- and SUV_max_-voxels was 14.0 mm (average of two readers). Spatial mismatch (*D* > 12 mm) between ADC_min_ and SUV_max_ was found in 9/25 patients. A considerable number of mismatch cases (65%) was also seen in a control group that underwent simultaneous PET/MRI. In the entire patient cohort, no statistically significant correlation between SUV_max_ and ADC_min_ was seen, while a moderate negative linear relationship (*r* = −0.5) between SUV_max_ and ADC_min_ was observed in tumors with a spatial match (*D* ≤ 12 mm). In conclusion, spatial mismatch between ADC_min_ and SUV_max_ is found in a considerable percentage of patients. The spatial connection of the two parameters SUV_max_ and ADC_min_ has a crucial influence on their numeric correlation.

## 1. Introduction

Hybrid ^18^F-fluorodeoxyglucose positron emission tomography/computed tomography (FDG-PET/CT) is an established method for assessment of lung tumors and is the current standard of reference for noninvasive preoperative staging of non-small-cell lung cancer (NSCLC) [1, 2]. An important strength of FDG-PET/CT in this context is its ability to quantify the glucose uptake within a lesion of interest by means of the standardized uptake value (SUV), using its maximum value (SUV_max_) within a lesion as a marker of tumor aggressiveness [3].

Diffusion-weighted magnetic resonance imaging (DW-MRI) and its derivative whole-body diffusion-weighted imaging with background signal suppression (DWIBS) [4] have emerged as an additional technology for functional assessment of solid tumors using the apparent diffusion coefficient (ADC) as a quantitative measure [5]. The ADC is a parameter that reflects the Brownian movement of water molecules and is linked to cell density, microvascular circulation, and membrane integrity in tumors [3].

Previous studies have extensively investigated the relationship between ADC and SUV, resulting in conflicting results. Recently, a meta-analysis including a subanalysis of 10 studies with a focus on lung tumors has calculated a pooled *r* of −0.35 and significant heterogeneity among the studies [3]. Because of this inverse behavior, some authors suggest the minimum ADC value (ADC_min_) found within a region of interest (ROI) as a substitute for the SUV_max_ when characterizing the aggressiveness or malignancy of a particular lesion [6–8]. On the other hand, other authors suggest that the parameters offer complementary information [3].

In their paper from 2013, Rose et al. scrutinized the concept of ADC_min_ measurements showing that there was minimal anatomic overlap between regions exhibiting minimum ADC and maximum uptake at ^18^F-fluoro-L-dopa PET in newly diagnosed gliomas [9]. We therefore set out to evaluate the numeric correlation of the two parameters as a function of their spatial relationship in sequential PET/CT plus MRI and simultaneous PET/MRI of lung tumor patients. Such an elucidation beyond the numeric correlation of the two parameters is important for a further understanding of their complex interplay.

## 2. Materials and Methods

### 2.1. Sequential PET/MRI

#### 2.1.1. Patients

Data from 25 patients (16 men, 9 women, mean age 67 years, and age range 45–84 years) were evaluated retrospectively. The patients had been enrolled in a prospective study that compared the diagnostic performance of FDG-PET/CT and MRI including diffusion-weighted imaging for preoperative staging [10]. All patients underwent PET/CT and whole-body MRI including DW-MRI before surgery. The predominant histological subtypes of pulmonary malignancies according to the WHO classification [11] were adenocarcinoma (16 cases) and squamous cell carcinoma (4 cases). Other present types of pulmonary malignancy were adenosquamous carcinoma (1 case), large cell carcinoma (2 cases), neuroendocrine tumor (1 case), and small cell carcinoma (1 case). The mean time interval between PET/CT and MRI was 15 days with a range from −1 to 29 days. No therapy was performed between the PET/CT and MRI examinations.

All procedures were in accordance with the Declaration of Helsinki (1964) and written informed consent was obtained from all patients. The requirements of the Institutional Ethical Committee were fulfilled.

#### 2.1.2. Imaging Protocol

PET/CT examinations were performed on an integrated PET/CT system with 16-slice CT (Discovery STE, GE Healthcare, Chalfont St Giles, UK). A mean dose of 353 ± 72 MBq ^18^F-FDG was administered 60 min before imaging after a fasting period of at least 6 h. For PET acquisition, 8 bed positions with each 4 min data acquisitions were obtained from skull to upper thigh. PET images were generated using 3D iterative OSEM reconstruction (image matrix, 128 × 128; voxel size 5.0 × 5.0 × 3.27 mm, 3 iterations, 35 subsets, and Gaussian filter with 5.49 mm FWHM). All patients received unenhanced low dose CT for attenuation correction and anatomical reference (tube voltage = 120 kV, tube current = 100 mA, collimation = 16 × 3.75 mm, and shallow breathing).

MRI examinations were performed on a 1.5-T whole-body MRI (Magnetom Avanto or Magnetom Symphony, Siemens Healthineers, Erlangen, Germany) using a dedicated 18-channel coil array system. DWIBS sequences were applied with the following parameters: single shot echo planar imaging [ss-EPI], TR = 5400 ms, TE = 58 ms, *b* = 0 and 800 s/mm^2^, STIR fat suppression with TI = 180 ms, matrix size = 192 × 144 pixels, slice thickness = 5 mm, voxel size 2.6 × 2.6 × 5 mm^3^, 4 averages, acquisition time 7 × 1:43 min, transverse orientation, and 7 acquisition steps from skull to upper thigh. All data were acquired during shallow breathing. No contrast agent was applied.

#### 2.1.3. Image Analysis

The fusion registration of the FDG-PET and DW-MRI data was performed by two board certified radiologist and nuclear medicine physicians with 10 years' (reader 1, GS) and 9 years' (reader 2, AS) experience in MRI and PET/CT reading on a commercially available workstation (Syngo MMWP, Software Version VE60A, Siemens Healthineers, Erlangen, Germany). The two readers performed manual rigid body translations in 3 dimensions to match the tumor outlines on the PET and DW-MRI (*b* = 800 s/mm^2^) datasets visually and then recorded the coordinates of the 3D translation vectors for all patients.

The PET data were evaluated by reader 1 using a commercially available workstation (SyngoVia, Software Version 3.0, Siemens Healthineers, Erlangen, Germany). The lesions were segmented semiautomatically using a dedicated 3D volume of interest (VOI) segmentation tool and an adaptive thresholding method as published by Brambilla et al. [12]. With this approach, their SUV_max_ and SUV_mean_ values were recorded and, in addition, the metabolic tumor volume (*V*_PET_), the maximum diameter of the lesion in 3 dimensions (*D*), and the coordinates of the SUV_max_-voxel were measured.

**Figure 1:**
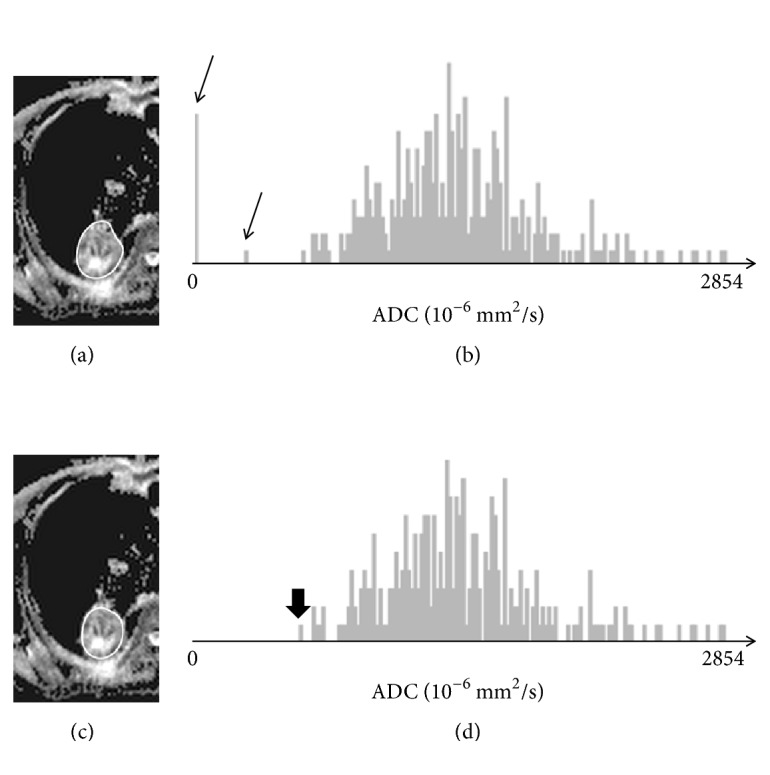
Refining of ROIs avoiding low-value outliers using online histogram analysis. (a) Original ROI drawn manually on the DWI *b* = 800 dataset and copied to the ADC map. (b) Histogram of the original ROI showing low ADC outliers (arrows). (c-d) ROI and histogram after correction with removed outliers and “true” ADC_min_ indicated (bold arrow).

The DW-MRI data were evaluated using Osirix Lite 7.5.1 (Pixmeo SARL, Bernex, Switzerland). This was done by reader 1 and by a less experienced reader (reader 3, TW), a resident in radiology with 1 year of professional experience. The tumor outlines were segmented manually as stacks of 2D ROIs on the *b* = 800 s/mm^2^ DW images and were copied subsequently onto the ADC maps. The ROIs were then refined manually on the ADC maps to avoid any low-value outliers to the pixel distribution using an online histogram analysis of each ROI as the reference (see Figure 1). As outcome parameters, the tumor volume *V*_MRI_, ADC_mean_, ADC_min_, and the coordinates of the ADC_min_-voxel were recorded.

In the final step of the image evaluation, the PET images and ADC maps were read side by side to assess the ADC at the position of the SUV_max_ and vice versa. Readers 1 and 3 did this by applying the 3D translation vectors from the fusion registration process to convert the coordinates between the PET and MRI frames of reference. The measured outcome parameters were the ADC at the position of the SUV_max_ (ADC@SUV_max_), the mean and minimum ADC in a 1 cm^3^ sphere around the position of the SUV_max_ (ADC_min_@SUV_max_ and ADC_mean_@SUV_max_) and, accordingly, the SUV at the position of the ADC_min_ (SUV@ADC_min_), and the mean and maximum SUV in a 1 cm^3^ sphere around the position of the ADC_min_ (SUV_max_@ADC_min_ and SUV_mean_@ADC_min_).

Amira 5.4.5 (Zuse Institute, Berlin, Germany and FEI Visualization Sciences Group, Bordeaux, France) was used for 3D visualization of patient cases.

### 2.2. Simultaneous PET/MRI

#### 2.2.1. Patients

Data from 10 patients (7 men, 3 women, mean age 62 years, and age range 38–73 years) were evaluated retrospectively. The patients had been enrolled in a previous study that assessed the diagnostic performance of simultaneous whole-body PET/MRI in patients with suspected lung cancer [13]. All patients underwent clinically indicated FDG-PET/CT and additional subsequent whole-body PET/MRI at the same day.

All procedures were in accordance with the Declaration of Helsinki (1964) and written informed consent was obtained from all patients. The requirements of the Institutional Ethical Committee were fulfilled.

#### 2.2.2. Imaging Protocol

All patients had fasted for at least 6 hours before and had blood glucose levels in the reference range. After the intravenous injection of mean 350 ± 20 MBq ^18^F-FDG the patients underwent a standard whole-body PET/CT scan (Hi-Rez Biograph 16 or Biograph mCT; Siemens Healthineers, Knoxville, USA) followed by a simultaneous whole-body PET/MRI examination (Biograph mMR; Siemens Healthineers, Erlangen, Germany). Positron emission tomography and MR imaging were started simultaneously 123 ± 8.4 minutes p.i., with 6 minutes per bed. The PET data were reconstructed with an iterative 3D OSEM algorithm using 3 iterations and 21 subsets and a 3-mm Gaussian filter (image matrix, 256 × 256; voxel size, 1.78 × 1.78 × 2 mm). The PET attenuation correction was accomplished by a MR-based attenuation map from segmented MR images. During each PET data acquisition, axial ss-EPI sequences were acquired under free breathing with the following parameters: TR = 13300 ms, TE = 76 ms, *b* = 0 and 800 s/mm^2^, STIR fat saturation, matrix size = 104 × 138 pixels, voxel size 2.8 × 2.8 × 6.0 mm^3^, 3 averages, parallel imaging acceleration factor = 2, and acquisition time 3:20 min per bed.

#### 2.2.3. Image Analysis

For simultaneous PET/MRI, readers 1 and 3 evaluated the PET and MRI data subsequently by measuring the coordinates of the SUV_max_- and ADC_min_-voxel and other parameters as described above.

### 2.3. Statistical Analysis

Statistics were calculated with JMP Version 12.2 (SAS Institute Inc., Cary, NC, USA). All data are reported as mean ± standard deviation (SD). Pearson's product-moment correlation coefficient (*r*_*P*_) and Spearman's rank correlation coefficient (*r*_*S*_) were determined for correlation analysis. Unpaired *t*-test was chosen for the comparison of parameters. For all tests, *P* values smaller than 0.05 were considered statistically significant.

## 3. Results

### 3.1. Sequential PET/MRI

#### 3.1.1. Accuracy of Manual PET and DWI Registration

The mean registration difference of PET and *b* = 800 DW-MRI between readers 1 and 2 was 6.0 ± 3.5 mm (range: 0.7–14.3 mm). This resulted in an upper limit of the 95% confidence interval for registration accuracy of 12 mm. This value of 12 mm was used in the following as a threshold to describe a spatial match of SUV_max_ and ADC_min_ (if the distance between their voxels was ≤12 mm) or otherwise a spatial mismatch of the two parameters (if the distance between the voxels was >12 mm).

#### 3.1.2. Spatial Correlation of SUV_max_ and ADC_min_

The mean distance between the SUV_max_- and ADC_min_-voxel was 12.7 ± 8.7 mm for reader 1 and 15.2 ± 10.5 mm for reader 3. Using the above-mentioned threshold of 12 mm, a spatial match between ADC_min_- and SUV_max_-voxels was found in 17/25 cases (68%) for reader 1 and in 15/25 cases (60%) for reader 3, whereas a spatial mismatch was seen in the remaining cases (8 (32%) for reader 1, 10 (40%) for reader 3). All tumors with spatial mismatch were larger than 3 cm, except for one case evaluated by reader 3 (tumor diameter 26 mm, distance between SUV_max_- and ADC_min_-voxels 16 mm).

**Figure 2:**
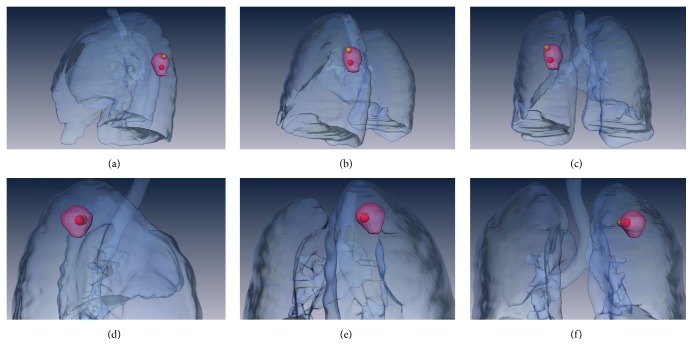
3D visualization of patient cases with spatial mismatch and match between ADC_min_ and SUV_max_. (a–c) Patient with adenocarcinoma in the left lower lobe superior segment in lateral (a), posterior oblique (b), and posterior (c) projections. (d–f) Patient with adenocarcinoma in the right upper lobe apical segment in lateral (a), posterior oblique (b), and posterior (c) projections. The orange points indicate the ADC_min_ and the red points the SUV_max_. The distance between both parameters in the first patient is 19.5 mm and 5.3 mm in the second patient.

A typical example of an adenocarcinoma in the right upper lobe apical segment with spatial mismatch (distance = 19.5 mm) between ADC_min_ (orange point) and SUV_max_ (red point) is represented in Figures 2(a)–2(c) as 3D visualization (see also supplemental Video 1). Figures 2(d) and 2(e) show an adenocarcinoma in the right upper lobe apical segment with ADC_min_ (orange point) and SUV_max_ (red point) located nearby (distance = 5.3 mm) (see also supplemental Video 2).

#### 3.1.3. Tumor Volume

**Figure 3:**
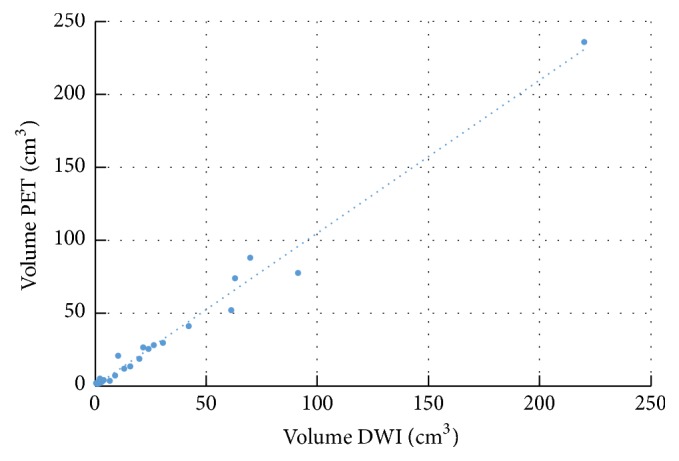
Correlation between PET- and DWI-derived tumor volumes.

The PET-derived tumor volume was 31.2 ± 49.7 cm^3^ (mean ± SD). The ADC-derived volume was 29.8 ± 46.9 cm^3^ for both readers. An almost perfect correlation between both volumes was observed (reader 1: *r*_*P*_ = 0.99, *P* < 0.0001, *r*_*S*_ = 0.97, Figure 3; reader 3: *r*_*P*_ = 0.98, *P* < 0.001,  *r*_*S*_ = 0.92). There was a statistically significant difference between patients with spatial match and patients with spatial mismatch in terms of tumor diameter for reader 1 (39.3 ± 22.0 mm versus 77.1 ± 34.6 mm, *P* = 0.003), whereas the difference was not significant for reader 3 (41.7 ± 25.8 mm versus 66.0 ± 35.0 mm, *P* = 0.057).

#### 3.1.4. SUV and ADC Measurements

Table 1 displays the average SUVs and ADC values of all tumors and the following subgroups: spatial mismatch (all; diameter > 3 cm), spatial match (all), spatial match (diameter < 3 cm), and spatial match (diameter > 3 cm). For reader 3, there was a statistically significant difference in average ADC_min_ between tumors with spatial match and spatial mismatch (*P* = 0.007), which was not the case for reader 1 (*P* = 0.38). Otherwise, no statistically significant differences between the parameters in the spatial mismatch and match groups were found, except for the mean distance between SUV_max_ and ADC_min_ and the tumor diameter. There was an almost perfect positive correlation between SUV_max_ and SUV_mean_ (Table 2 and supplemental Figure 1) and a strong positive correlation between ADC_min_ and ADC_mean_ (Table 2 and supplemental Figures 1 and 2), when considering all tumors.

#### 3.1.5. Numeric Correlation of SUV_max_ and ADC_min_

**Figure 4:**
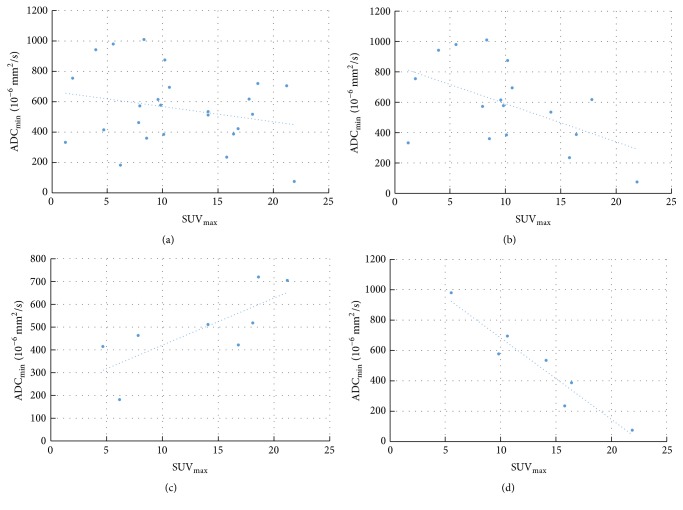
Correlation between SUV_max_ and ADC_min_: all tumors (a), tumors with spatial match (b), tumors with spatial mismatch (c), and tumors > 3 cm with spatial match (d).

The results of the numeric correlation are given in Table 2. In the entire cohort, no significant correlation was seen between SUV and ADC values SUV_max_/ADC_min_ (Figure 4(a)), SUV_mean_/ADC_mean_, SUV_max_/ADC_mean_@SUV_max_, and ADC_min_/SUV_mean_@ADC_min_.

In the spatial match group, the correlation analysis disclosed a moderate inverse correlation between SUV_max_/ADC_min_ for reader 1 (Figure 4(b)) and ADC_min_/SUV_mean_@ADC_min_ for reader 3. No significant correlation was seen for the other pairs of parameters.

**Figure 5:**
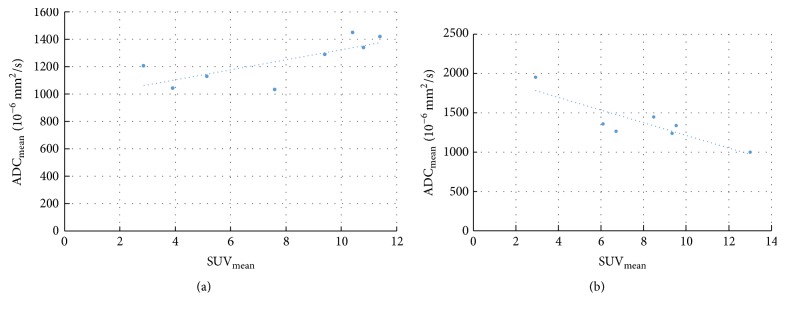
Correlation between SUV_mean_ and ADC_mean_ in tumors > 3 cm: tumors with spatial mismatch (a) and tumors with spatial match (b).

In the spatial mismatch group, the correlation analysis of reader 1 disclosed a strong positive correlation between SUV_max_/ADC_min_ (Figure 4(c)) and SUV_mean_/ADC_mean_ (Figure 5(a)), while reader 3 revealed no significant correlations in both cases. No significant correlations were seen by both readers for SUV_max_/ADC_mean_@SUV_max_ and ADC_min_/SUV_mean_@ADC_min_.

Ten patients had a tumor diameter of ≤3 cm, corresponding to a tumor stage T1. All of them showed a spatial match between SUV_max_ and ADC_min_, except for one case evaluated by reader 3. Correlation analysis between SUV and ADC values disclosed no significant linear correlations for SUV_max_/ADC_min_, SUV_mean_/ADC_mean_, SUV_max_/ADC_mean_@SUV_max_, and ADC_min_/SUV_mean_@ADC_min_.

Fifteen patients had a tumor diameter > 3 cm (tumor stages T2–T4). These were almost equally subdivided into a spatially matched (reader 1: *n* = 7, reader 3: *n* = 6) and a spatially mismatched subgroup (*n* = 8 and *n* = 9, respectively). Correlation analysis between SUV and ADC values in the matched group disclosed an almost perfect inverse correlation between SUV_max_/ADC_min_ (Figure 4(d)), SUV_mean_/ADC_mean_ (Figure 5(b)), SUV_max_/ADC_mean_@SUV_max_, and ADC_min_/SUV_mean_@ADC_min_. However, the *P* values for the correlations of SUV_max_/ADC_min_ and SUV_max_/ADC_mean_@SUV_max_ for reader 3 were slightly above the 0.05 threshold for statistical significance. In the mismatch subgroup a weak to moderate positive correlation was seen for any pair of parameters. Here, statistical significance was reached only for reader 1 and the pairs SUV_max_/ADC_min_ and SUV_mean_/ADC_mean_.

### 3.2. Interobserver Agreement

Supplemental Figure 3(1) elucidates the differences between readers 1 and 3, plotted against the averages of the distances between SUV_max_ and ADC_min_ for the sequential PET/MRI measurements. The analysis indicates that 6/25 measurements are outside the 95% confidence interval for both the upper and lower limits of agreement. From these, 5 distance measurements were grouped in different categories (match versus mismatch) by the two readers. When reading the absolute ADC_min_ values, 7/25 measurements were outside the 95% confidence interval (supplemental Figure 3(2)).

### 3.3. Simultaneous PET/MRI Imaging

Table 3 summarizes the SUV, ADC values, mean distance of SUV_max_ and ADC_min_, and the tumor diameter of the patients examined with simultaneous PET/MRI.

The mean distance between the ADC_min_- and SUV_max_-voxels was 22.6 ± 18.1 mm. In 4 tumors the distance was <10 mm, while for the remaining 6 tumors the distance was >20 mm. All tumors with spatial mismatch were larger than 4 cm. There was only 1 tumor with spatial match of SUV_max_/ADC_min_ and a size of more than 3 cm.

The results of the numeric correlations are shown in supplemental Table 1. No significant correlation was seen in the entire dataset for SUV_max_/ADC_min_, SUV_mean_/ADC_mean_, SUV_max_/ADC_mean_@SUV_max_, and ADC_min_/SUV_mean_@ADC_min_. The results of the subgroup analyses also provided no statistically significant results.

## 4. Discussion

Numerous studies using sequential [14, 15] or simultaneous [13, 16, 17] PET/MRI and including all histological subtypes have compared ADC and SUV values in lung tumors and reported an inverse numeric correlation of the two parameters. Three studies showed no numeric correlation [3, 18–20]. The work in hand is, to the best of our knowledge, the first study that analyzes ADC_min_ and SUV_max_ data with regard to their spatial correlation. As shown in our study, spatial mismatch occurred in almost one-third of the tumors examined with sequential PET/MRI (7/25) and in every second tumor larger than 3 cm (8/15). Somewhat surprisingly, the rates of spatial mismatch were even higher in our small control sample of 10 tumors examined with simultaneous PET/MRI, where 7/10 tumors overall and 6/7 tumors > 3 cm showed a spatial distance > 20 mm between the SUV_max_- and ADC_min_-voxels. Thus, the spatial mismatch between the two regions in the sequential part of the study is unlikely to be related in full to the sequential nature of the PET/CT plus MRI set-up.

The two subgroups with spatial match (distance SUV_max_ - ADC_min_ ≤ 12 mm) and mismatch (distance > 12 mm) were defined using a threshold of 12 mm based on the measured accuracy of the spatial registration of the sequential PET and MRI datasets. This threshold definition is in concordance with a previous study of Rakheja et al. that compared the accuracy of the spatial registration between PET/CT and simultaneous PET/MRI and calculated a registration difference of 6.61 ± 1.6 mm between DWI and PET in a subgroup of 6 lung lesions [21]. The authors of that article explain this by the inherent spatial distortion of EPI sequences linked to eddy currents and nonlinearities of the gradient coils [22]. In another study, Schmidt et al. reported a slightly larger mean cumulative misalignment of 7.7 mm between DWI and PET [13]. This uncertainty stemming from spatial coregistration mainly affects tumors of smaller size in our study, that is, the subgroup of tumors < 3 cm. In these tumors, it cannot be said with reasonable certainty whether the observed spatial differences between SUV_max_ and ADC_min_ are caused by an actually underlying mismatch or by registration inaccuracy. Also any existing spatial mismatch that ranges below the limit set by the registration accuracy cannot be detected. In line with these considerations, our pooled data of the sequential PET/MRI patient cohort indicate no significant correlation between SUV and ADC values (SUV_max_/ADC_min_, SUV_mean_/ADC_mean_, SUV_max_/ADC_mean_@SUV_max_, ADC_min_/SUV_mean_@ADC_min_), while a strong and significant linear correlation between SUV_max_ and ADC_min_ is seen for tumors > 3 cm, whereby the direction (positive versus negative) depends on the spatial relationship of the two parameters.

While the lack of a significant correlation of SUV_max_ and ADC_min_ in our total patient sample is in accordance with at least some of the previous studies [3, 18–20], the finding of a size-dependent spatial mismatch of the two parameters that affects their numeric correlation has—to the best of our knowledge—not been reported before. It is known, however, that anatomic tumor size and heterogeneity in lung cancer, for example, represented by FDG uptake, are intimately connected with each other [23]. Hence, one may hypothesize that the increase in distance between ADC_min_- and SUV_max_-voxels with increasing tumor diameter that is observed in our study may at least partly be an effect of increasing heterogeneity. While most of the above-mentioned studies that analyzed the relationship between ADC_min_ and SUV_max_ did not comment on tumor size as a contributor to heterogeneity [13, 14, 16, 17], tumor size (mean diameter) is reported only in two of them (4.9 cm (range 2.4–13.7 cm) in [24]; 5.9 cm (range 4–10 cm) in [19]). The mean tumor diameter of approx. 5 cm in our study is comparable with these two studies. It may be assumed that, in the other studies, more tumors of smaller size or with matching areas of SUV_max_ and ADC_min_ have been included resulting in inverse correlations within whole groups.

One limitation of the previous studies is that the ADC_min_ is derived from a single voxel, potentially introducing a sampling bias. This problem also becomes evident in our own study, where considerable differences in the measurement of ADC_min_ occurred between the two readers with different levels of experience. As a potential solution to this problem, Gong et al. proposed thresholding of DWI histograms using a *k*-means clustering algorithm and presumption of 3 tissue classifications [25]. With this approach, they showed a stronger correlation between the threshold-based ADC and SUV_max_ (*r* = −0.843), compared to gross ADC and SUV_max_ (*r* = −0.739) for gastrointestinal stromal tumors. The authors discuss that the segmentation of high-cellularity tissues matches better to hypermetabolic tissues. An interesting approach was also followed by Metz et al. discriminating peripheral from central tumor regions with defined thresholds, voxel-by-voxel correlation and cluster analysis in a few NSCLC patients [19]. In their work, the tumor area was divided into four regions with different assumed “biological activity”: SUV_high_/ADC_high_ (cell edema, micronecrosis, hypoxia), SUV_high_/ADC_low_ (viable tumor), SUV_low_/ADC_high_ (necrosis), and SUV_low_/ADC_low_ (hibernating tumor cells, desmoplastic reaction). So, for instance, 72% of all voxels with low ADC were located in the SUV_high_ cluster, while 83% of the SUV_low_ cluster voxels, mainly located in the tumor center, also showed lower ADC values. In accordance with these results, also our data suggest that the two markers, SUV and ADC, reflect different tissue properties that are subject to biological changes and are not necessarily in a linear correlation. This finding is particularly interesting in the dawning era of radiomics, as it may support the development of more specific imaging biomarker profiles of tumors and help improving the ability of multiparametric models to predict response to therapy and survival. Variation in ADC may also stem from scanner settings and ROI definitions. While some groups suggest to combine *b*  =  0 images and contrast-enhanced T1-weighted images [16] for the definition of tumor ROIs, others refer to *b* = 800 images [26]. A few studies have addressed repeatability and found in general good interobserver repeatability for the mean ADC at 1.5 T [27] and 3 T [26]. Regier et al. documented an intraclass correlation coefficient of 0.88 at 1.5 T [14]; others report a value of 0.92 for ADC_min_ at 3 T [24]. However, when comparing ADC histograms in previous studies, it becomes evident that some have included 0 values that might result from pneumatized lung tissue [19], while others have truncated low ADC values by thresholding [13]. We therefore believe that our approach using VOI histogram verification yields more consistent results for the identification of intratumoral ADC_min_ than the methods used in many of the previous studies.

Likewise, SUV measurements can be influenced by a variety of biologic and technologic factors [28]. The different FDG uptake time, attenuation correction, and scanner resolution result in higher SUV_mean_ and SUV_max_ values of lung lesions measured using PET/MRI compared to PET/CT [29]. Being aware of these effects, we abstained from including the results from simultaneous PET/MRI into the numeric analysis, in order to avoid mixing SUV data stemming from different machines and acquired at different time points.

This study has several other potential limitations. First to mention is the small patient number that makes our data susceptible to selection bias. In particular, the results of the subgroup analyses (*n* = 8, 10, 15, and 17) have limited statistical robustness and require corroboration by further studies of larger cohorts. However, with 25 included patients overall, our study ranges in the same order of magnitude as several previous studies (*n* = 41 [14], *n* = 15 [17], *n* = 18 [16], *n* = 15 [13], *n* = 36 [15]). Another limitation of the study design is the temporal delay between PET/CT and MRI within the sequential PET/MRI approach, which has been limited to 1 month. Nevertheless, PET- and ADC-derived tumor volumes match very well, as evident from Figure 3, and therefore no significant growth between both measurements was documented. Also, a recent meta-analysis has defined a threshold interval of one month as inclusion criterion [3]. Additionally, the image resolutions of the two different modalities are different and subsequently ADC and SUV data differ in pixel size. Finally, histopathological correlation could not be performed because of missing spatial allocation between pathology and imaging.

## 5. Conclusions

In conclusion, spatial mismatch between VOI-based ADC_min_ and SUV_max_ is found in a considerable percentage of lung tumors and has a critical influence on the numeric correlation of the two parameters: In our study, the significant negative correlation between SUV_max_ and ADC_min_ that has been reported by many previous studies is only seen in tumors > 3 cm without spatial mismatch. These results suggest that the information contained in the two parameters SUV and ADC is not interchangeable but reflects different tissue properties that may be combined to a more specific imaging biomarker profile of tumors.

## Figures and Tables

**Table 1:** Average standardized uptake values and intratumoral ADC values of the groups analyzed with sequential PET/MRI (mean ± SD). Values are given separately for both readers: maximum standardized uptake value (SUV_max_), mean standardized uptake value (SUV_mean_), minimum apparent diffusion coefficient (ADC_min_), and mean apparent diffusion coefficient (ADC_mean_).

Reader 1	All tumors (*n* = 25)	Spatial mismatch (all diameters > 3 cm, *n* = 8)	Spatial match (all, *n* = 17)	Spatial match (diameter < 3 cm, *n* = 10)	Spatial match (diameter > 3 cm, *n* = 7)
SUV_max_	11.2 ± 5.9	13.4 ± 6.3	10.2 ± 5.6	8.0 ± 4.8	13.5 ± 5.3
SUV_mean_	6.8 ± 3.3	7.7 ± 3.3	6.1 ± 3.3	4.8 ± 2.8	8.0 ± 3.2
ADC_min_ (10^−6^ mm^2^/s)	555 ± 244	492 ± 172	585 ± 271	647 ± 245	497 ± 300
ADC_mean_ (10^−6^ mm^2^/s)	1340 ± 252	1239 ± 162	1387 ± 276	1398 ± 279	1372 ± 293
Mean distance SUV_max_ and ADC_min_ (mm)	12.7 ± 8.7	22.4 ± 9.0	8.2 ± 3.1	7.5 ± 3.0	9.0 ± 3.4
Mean tumor diameter (mm)	51.4 ± 31.5	77.1 ± 34.6	39.3 ± 22.0	25.8 ± 2.2	58.7 ± 23.2

Reader 3	All tumors (*n* = 25)	Spatial mismatch (*n* = 10)	Spatial match (all, *n* = 15)	Spatial match (diameter < 3 cm, *n* = 9)	Spatial match (diameter > 3 cm, *n* = 6)

SUV_max_	11.2 ± 5.9	13.1 ± 5.6	10.0 ± 6.0	6.9 ± 3.5	14.8 ± 5.9
SUV_mean_	6.6 ± 3.3	7.6 ± 3.0	6.2 ± 3.4	4.2 ± 2.1	8.7 ± 3.5
ADC_min_ (10^−6^ mm^2^/s)	494 ± 268	325 ± 140	607 ± 277	679 ± 240	498 ± 315
ADC_mean_ (10^−6^ mm^2^/s)	1378 ± 345	1225 ± 129	1480 ± 406	1433 ± 312	1550 ± 544
Mean distance SUV_max_ and ADC_min_ (mm)	15.2 ± 10.5	25.5 ± 9.2	8.3 ± 3.0	7.6 ± 3.0	9.3 ± 2.9
Mean tumor diameter (mm)	51.4 ± 31.5	66.0 ± 35.0	41.7 ± 25.8	25.8 ± 2.3	65.6 ± 26.8

**Table 2:** Correlation coefficients and *P* values for sequential PET/CT and DW-MRI. Statistically significant correlations (*P* < 0.05) are indicated in bold. Maximum standardized uptake value (SUV_max_), mean standardized uptake value (SUV_mean_), minimum apparent diffusion coefficient (ADC_min_), and mean apparent diffusion coefficient (ADC_mean_).

	Reader 1			Reader 3		
	*r*_*P*_	*P*	*r*_*S*_	*r*_*P*_	*P*	*r*_*S*_
*All tumors*	*n = 25*			*n = 25*		
Vol_PET_/Vol_MRI_	**0.99**	**<0.001**	0.97	**0.98**	**<0.001**	0.92
SUV_max_/SUV_mean_	**0.99**	**<0.001**	0.98	—	—	—
ADC_min_/ADC_mean_	**0.74**	**<0.001**	0.69	**0.61**	**0.001**	0.62
SUV_max_/ADC_min_	−0.25	0.24	−0.12	−0.38	0.06	−0.32
SUV_mean_/ADC_mean_	−0.11	0.61	−0.02	−0.19	0.37	−0.03
SUV_max_/ADC_mean_@SUV_max_	−0.15	0.49	−0.13	—	—	—
ADC_min_/SUV_mean_@ADC_min_	−0.26	0.21	−0.12	−0.32	0.12	−0.27

*Spatial match*	*n = 17*			*n = 15*		
SUV_max_/ADC_min_	−**0.52**	**0.03**	−0.39	−0.48	0.07	−0.36
SUV_mean_/ADC_mean_	−0.26	0.31	−0.18	−0.19	0.49	−0.05
SUV_max_/ADC_mean_@SUV_max_	−0.26	0.30	−0.31	−0.22	0.42	−0.25
ADC_min_/SUV_mean_@ADC_min_	−0.39	0.12	−0.18	−**0.54**	**0.04**	−0.26

*Spatial mismatch*	*n = 8*			*n = 10*		
SUV_max_/ADC_min_	**0.76**	**0.02**	0.88	0.27	0.45	0.32
SUV_mean_/ADC_mean_	**0.75**	**0.02**	0.71	0.32	0.36	0.45
SUV_max_/ADC_mean_@SUV_max_	0.56	0.14	0.67	0.35	0.32	0.34
ADC_min_/SUV_mean_@ADC_min_	0.60	0.10	0.55	0.29	0.40	0.05

*Diameter ≤ 3 cm*	*n = 10*			*n = 10*		
SUV_max_/ADC_min_	−0.01	0.97	0.10	−0.31	0.37	−0.13
SUV_mean_/ADC_mean_	0.20	0.57	0.26	−0.03	0.94	0.09
SUV_max_/ADC_mean_@SUV_max_	0.06	0.87	0.02	—	—	—
ADC_min_/SUV_mean_@ADC_min_	0.11	0.77	0.30	0.07	0.85	0.04

*Diameter > 3 cm & spatial match*	*n = 7*			*n = 6*		
SUV_max_/ADC_min_	**−0.96**	**<0.001**	−0.93	−0.76	0.06	−0.60
SUV_mean_/ADC_mean_	**−0.87**	**0.006**	−0.75	**−0.81**	**0.03**	−0.54
SUV_max_/ADC_mean_@SUV_max_	**−0.75**	**0.04**	−0.5	−0.75	0.06	−0.60
ADC_min_/SUV_mean_@ADC_min_	**−0.76**	**0.04**	−0.71	**−0.85**	**0.02**	−0.89

*Diameter > 3 cm & spatial mismatch*	*n = 8*			*n = 9*		
SUV_max_/ADC_min_	**0.76**	**0.02**	0.88	0.39	0.29	0.41
SUV_mean_/ADC_mean_	**0.75**	**0.02**	0.71	0.59	0.09	0.67
SUV_max_/ADC_mean_@SUV_max_	0.56	0.14	0.67	0.53	0.14	0.61
ADC_min_/SUV_mean_@ADC_min_	0.60	0.10	0.55	0.33	0.39	0.13

**Table 3:** Standardized uptake values and intratumoral ADC values of the patients examined with simultaneous PET/MRI: maximum standardized uptake value (SUV_max_), mean standardized uptake value (SUV_mean_), minimum apparent diffusion coefficient (ADC_min_), and mean apparent diffusion coefficient (ADC_mean_). R1: reader 1; R3: reader 3.

Pat. nr.	SUV_max_	SUV_mean_	ADC_min_ (10^−6^ mm^2^/s)		ADC_mean_ (10^−6^ mm^2^/s)		Distance SUV_max_/ADC_min_ (mm)		Tumor diameter PET (mm)
			R1	R3	R1	R3	R1	R3	
(1)	22.6	13.4	451	334	1329	1296	20.2	21.8	74.5
(2)	7.2	4.3	6	6	1609	1584	56.5	56.5	96.4
(3)	14.4	8.4	12	12	1036	1016	28.7	28.7	93.6
(4)	8.5	4.6	553	222	1504	1612	48.0	33.0	85.6
(5)	6.1	3.7	235	564	1238	1430	4.8	4.0	29.2
(6)	9.5	5.9	422	381	1937	1339	3.1	8.6	24.0
(7)	8.0	4.8	779	575	1688	1694	9.5	12.6	34.2
(8)	19.7	11.5	49	49	1028	1052	24.8	24.8	54.4
(9)	8.1	4.9	553	638	1150	1164	7.7	3.9	20.4
(10)	2.6	1.4	228	337	1105	1121	22.9	22.4	46.9

*Average*	*10.7*	*6.3*	*329*	*312*	*1362*	*1331*	*22.6*	*21.6*	*55.9*

## Supplementary Materials

Supplementary 1 Supplemental Figure  1: correlation between SUV_max_ and SUV_mean_ in all tumors.

Supplementary 2 Supplemental Figure  2: correlation between ADC_min_ and ADC_mean_ in all tumors.

Supplementary 3 Supplemental Figure  3: Bland-Altman plots showing differences between readers 1 and 3 plotted against the averages of the distances between SUV_max_ and ADC_min_(1) and ADC_min_ values (2) for the sequential PET/MRI measurements.

Supplementary 4 Supplemental Video 1: 3D visualization of patient case with spatial mismatch between ADC_min_ and SUV_max_.

Supplementary 5 Supplemental Video  2: 3D visualization of patient case with spatial match between ADC_min_ and SUV_max_.

Supplementary 6 Supplemental Table  1: correlation coefficients and* P* values for simultaneous PET/MRI.
