# Supplementary figures and images for: The Spatial Relationship between Apparent Diffusion Coefficient and Standardized Uptake Value of 18F-Fluorodeoxyglucose Has a Crucial Influence on the Numeric Correlation of Both Parameters in PET/MRI of Lung Tumors
Source: Contrast Media Mol Imaging. 2017 Dec 17;2017:8650853. doi: 10.1155/2017/8650853 (PMC5748125; doi:10.1155/2017/8650853)

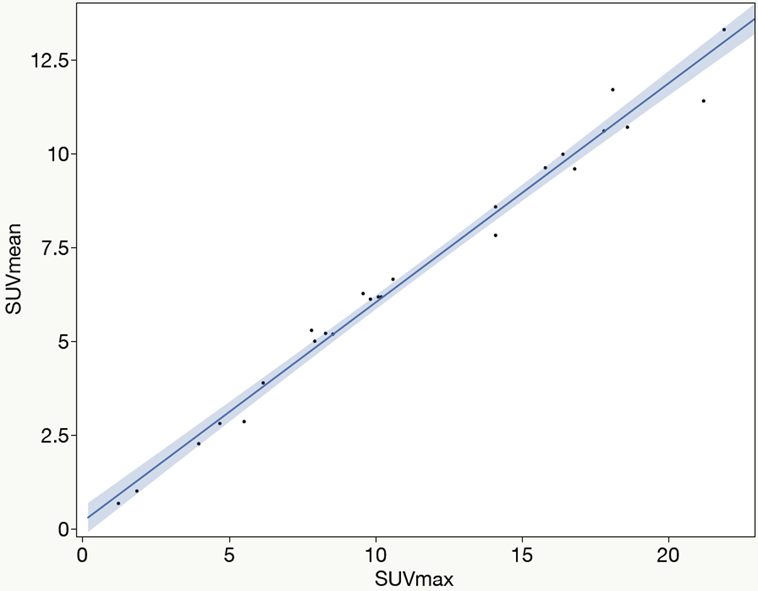

Supplement: Supplementary 1 — Supplemental Figure 1: correlation between SUVmax and SUVmean in all tumors. [file 8650853.f1.tif]

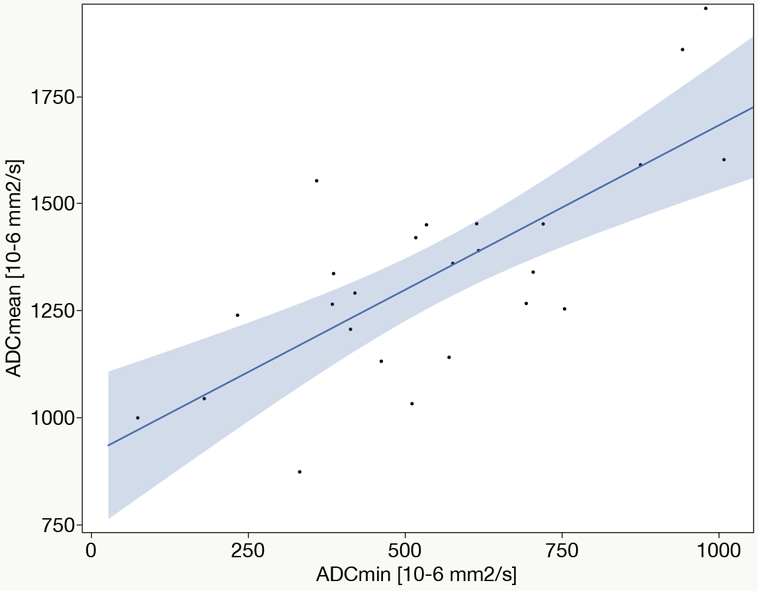

Supplement: Supplementary 2 — Supplemental Figure 2: correlation between ADCmin and ADCmean in all tumors. [file 8650853.f2.tif]

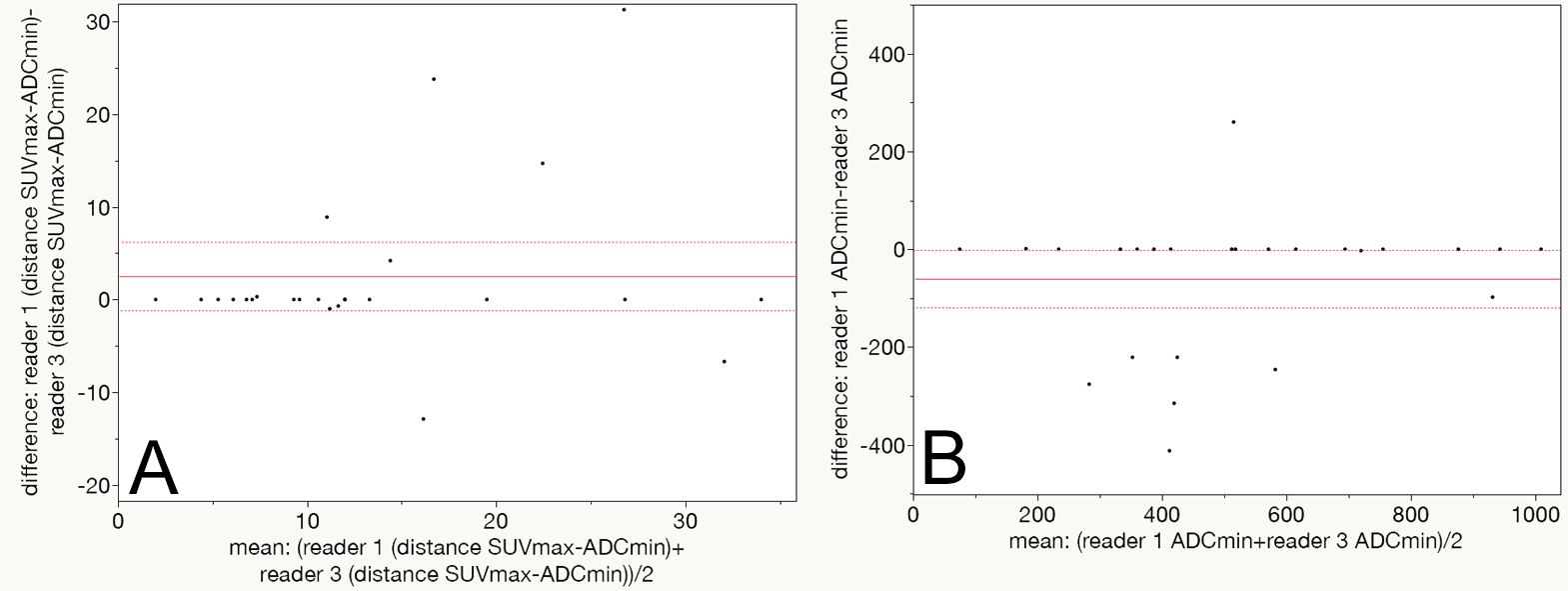

Supplement: Supplementary 3 — Supplemental Figure 3: Bland-Altman plots showing differences between readers 1 and 3 plotted against the averages of the distances between SUVmax and ADCmin(1) and ADCmin values (2) for the sequential PET/MRI measurements. [file 8650853.f3.tif]
