# Supplementary material for: The Spatial Relationship between Apparent Diffusion Coefficient and Standardized Uptake Value of 18F-Fluorodeoxyglucose Has a Crucial Influence on the Numeric Correlation of Both Parameters in PET/MRI of Lung Tumors
Source: Contrast Media Mol Imaging. 2017 Dec 17;2017:8650853. doi: 10.1155/2017/8650853 (PMC5748125; doi:10.1155/2017/8650853)
Supplement: Supplementary 6 — Supplemental Table 1: correlation coefficients and P values for simultaneous PET/MRI. [file 8650853.f6.pdf]

	**Reader 1**			**Reader 3**		
**All tumors**	n = 10			n= 10		
	**r_P**	**P**	**r_S**	**r_P**	**P**	**r_S**
VolPET/VolDWI	**0.90**	**<0.001**	0.82	**0.95**	**<0.001**	0.88
SUV_{max}/SUV_{mean}	**1.00**	**<0.001**	0.96	-	-	-
ADC_{min}/ADC_{mean}	0.46	0.18	0.41	0.24	0.50	0.25
SUV_{max}/ADC_{min}	-0.15	0.68	0.04	-0.37	0.29	-0.31
SUV_{mean}/ADC_{mean}	-0.21	0.55	-0.16	-0.35	0.31	-0.41
SUV_{max}/ADC_{mean}@SUV_{max}	0.12	0.72	0.19	-	-	-
ADC_{min}/SUV_{mean}@ADC_{min}	0.10	0.79	0.22	-0.09	0.80	-0.28
**Spatial match**	n = 4			n = 3		
	**r_P**	**P**	**r_S**	**r_P**	**P**	**r_S**
SUV_{max}/ADC_{min}	0.42	0.55	0.20	-0.61	0.50	-0.50
SUV_{mean}/ADC_{mean}	0.73	0.20	0.40	-0.39	0.70	-0.50
SUV_{max}/ADC_{mean}@SUV_{max}	0.04	0.96	0.00	-0.02	0.99	0.50
ADC_{min}/SUV_{mean}@ADC_{min}	0.59	0.36	0.60	-0.15	0.89	-0.50
**Spatial mismatch**	n = 6			n = 7		
	**r_P**	**P**	**r_S**	**r_P**	**P**	**r_S**
SUV_{max}/ADC_{min}	0.05	0.93	0.20	-0.23	0.62	-0.14
SUV_{mean}/ADC_{mean}	-0.27	0.60	-0.37	-0.38	0.38	-0.29
SUV_{max}/ADC_{mean}@SUV_{max}	0.25	0.62	0.26	0.19	0.68	0.11
ADC_{min}/SUV_{mean}@ADC_{min}	0.56	0.22	0.54	-0.02	0.97	-0.14
**Diameter <3 cm**	n = 3			n = 3		
	**r_P**	**P**	**r_S**	**r_P**	**P**	**r_S**
SUV_{max}/ADC_{min}	0.67	0.43	0.50	-0.61	0.50	-0.50
SUV_{mean}/ADC_{mean}	0.78	0.30	0.50	-0.39	0.70	-0.50
SUV_{max}/ADC_{mean}@SUV_{max}	-0.02	0.99	0.50	-	-	-
ADC_{min}/SUV_{mean}@ADC_{min}	0.61	0.49	0.50	-0.15	0.89	-0.50
**Diameter >3 cm**	n = 7			n = 7		
	**r_P**	**P**	**r_S**	**r_P**	**P**	**r_S**
SUV_{max}/ADC_{min}	-0.13	0.77	0.07	-0.23	0.62	-0.14
SUV_{mean}/ADC_{mean}	-0.34	0.44	-0.36	-0.38	0.38	-0.29
SUV_{max}/ADC_{mean}@SUV_{max}	0.19	0.68	0.11	-	-	-
ADC_{min}/SUV_{mean}@ADC_{min}	0.19	0.69	0.29	-0.02	0.97	-0.14

Supplemental table 1: Correlation coefficients and p-values for simultaneous PET/MRI. Statistically significant correlations ($p<0.05$) are indicated in bold. Maximum standardized uptake value (SUV_{max}), mean standardized uptake value (SUV_{mean}), minimum apparent diffusion coefficient (ADC_{min}), mean apparent diffusion coefficient (ADC_{mean}).
